# Apparent physical brightness of graphemes is altered by their synaesthetic colour in grapheme-colour synaesthetes

**DOI:** 10.1038/s41598-020-77298-2

**Published:** 2020-11-18

**Authors:** Kyuto Uno, Kazuhiko Yokosawa

**Affiliations:** Department of Psychology, The University of Tokyo, Tokyo, 113-0033 Japan

**Keywords:** Psychology, Human behaviour

## Abstract

Grapheme-colour synaesthesia is a condition in which the visual perception of letters or numbers induces a specific colour sensation. In this study, we demonstrated that the apparent physical brightness of graphemes is modulated by the synaesthetic colours elicited by them. Synaesthetes first selected a synaesthetic colour corresponding to each capital letter and digit. Then, we selected a grapheme stimulus with a bright synaesthetic colour and one with a dark colour for each synaesthete. Finally, synaesthetes and non-synaesthete controls participated in a brightness judgment task, in which each participant judged the real brightness of each of the two stimuli compared to a standard stimulus. Compared to non-synaesthetes, synaesthetes judged a grapheme with a bright synaesthetic colour to be brighter than one with a dark synaesthetic colour, suggesting that the synaesthetic colour experience of synaesthetes alters their brightness perception. Such alteration in real brightness perception was observed both in those who experienced synaesthetic colours in external space (projector-type synaesthetes) and in those who experienced such colours ‘in the mind’s eye’ (associator-type synaesthetes). These results support the view that early visual processing is modulated by feedback transmitted from the V4 colour area, the neural activation of which accompanies synaesthetic colour experience.

## Introduction

Synaesthesia is a condition in which the perception of a stimulus in one modality (the inducer) automatically triggers a secondary sensation in another modality or processing stream (the concurrent). The present study focused on grapheme-colour synaesthesia, in which the visual perception of letters or numbers (graphemes) induces a specific colour sensation (the synaesthetic colour). The associations between graphemes and colours vary widely among synaesthetes^1^, and are highly consistent over time within each synaesthete^2,3^.

When synaesthetes view a grapheme, they experience two colour sensations: the real colour of the grapheme and the synaesthetic colour associated with the grapheme. Although these two colour sensations are elicited as independent conscious experiences, many studies have demonstrated that these colours interfere with each other in cognitive tasks. For example, modified versions of the Stroop task revealed longer response times when participants named the real colour of graphemes that elicited incongruent rather than congruent synaesthetic colour experiences^4,5^, and when they named the synaesthetic colour of graphemes written in incongruent rather than congruent (similar) colours^6,7^.

The interaction between the synaesthetic colour associated with graphemes and the perception of brightness, as well as real colour, has been examined from a perceptual perspective. Two case studies first raised the possibility that synaesthetic colour sensation may be modulated by apparent illumination^8,9^. However, subsequent studies conducted on a larger number of synaesthetes have demonstrated that a synaesthetic colour elicited by a particular grapheme is constant regardless of a change in the real colour and luminance of a grapheme or its background^10–12^. Based on these findings, the mechanisms underlying synaesthetic colour processing are generally thought to be different from those underlying real colour and brightness perception^13^.

However, it remains generally unclear whether synaesthetic colours influence real colour and brightness perception, although evidence from neuroscience research supports this possibility. Synaesthetic colour experiences are believed to be accompanied by neural activation in the V4 visual areas^14–17^, which are implicated in normal colour vision^18^, although not all studies have found V4 activation in response to synaesthetic colour^19^. Some studies have raised the possibility that modulatory feedback is transmitted from V4 to the primary visual cortex^20,21^, and from the primary visual cortex to the lateral geniculate nucleus^20^. Although no studies have provided direct evidence that V4 activation with synaesthetic colour experience triggers modulatory feedback to the early visual pathway, it is quite possible that synaesthetic colour experiences modulate early visual processing.

To elucidate the mechanisms of the interaction between synaesthetic colour processing and real colour and brightness perception, we conducted an experiment to investigate whether the brightness of synaesthetic colours can modulate perception of real brightness. It has been suggested that each dimension of the lightness, chroma, and hue that define a synaesthetic colour is determined by the influence of different grapheme properties^22–24^. In particular, several studies^24–26^ have demonstrated that bright synaesthetic colours are elicited by those graphemes that are used more frequently. This suggests the presence of a strong relationship between grapheme properties and the lightness of synaesthetic colours. In addition, whereas it is unclear whether the chroma and hue of synaesthetic colours are independently affected by colour perception, psychophysical experiments have demonstrated that the brightness of synaesthetic colours is not affected by perceived brightness^11^. Based on these findings, we considered that an examination focusing on the brightness of synaesthetic colours was the best way to reveal the mechanism of the interaction between synaesthetic colour processing and normal visual perception.

If synaesthetic colour experience elicited by a grapheme modulates grapheme brightness perception, synaesthetes would perceive the brightness of a grapheme which elicits a bright synaesthetic colour as brighter than that of another grapheme which elicits a dark synaesthetic colour. To examine this possibility, we had each of the synaesthetes match both their subjective brightness of a grapheme with a bright synaesthetic colour and that with a dark synaesthetic colour to the subjective brightness of a standard stimulus by using the method of constant stimuli. We then compared the points of subjective equality.

We also examined whether the modulatory effect of synaesthetic colours on brightness perception differs depending on the differences in subjective experiences of synaesthetic colours (i.e., projectors versus associators). Projectors report experiencing synaesthetic colours in external space, whereas associators report experiencing synaesthetic colours ‘in the mind’s eye’, not in external space^6,27^. It has been shown that there are differences between projectors and associators in neural structure and functional connectivity related to the synaesthetic colour experience^28,29^. Rouw and Scholte^28^ found greater grey matter volume in projectors compared to associators in the modality-specific cortex (i.e., visual cortex, auditory cortex, motor cortex) and lower grey matter volume in hippocampus and parahippocampal gyrus, known for their role in memory. Using dynamic causal modelling for fMRI, Van Leeuwen et al.^29^ showed that V4 activation during synaesthesia was induced via a bottom-up pathway (direct connectivity from the visual word form area in fusiform gyrus) in projectors, but via a top-down pathway (via the parietal lobe) in associators. In addition, some studies indicate that the association between grapheme properties (e.g., shape, frequency, sound) and synaesthetic colours is different between projectors and associators^30,31^. Together, these studies suggest that differences in subjective experience of synaesthetic colours are related to the process by which synaesthetic colour experience is triggered. They indicate the possibility that the influence of the brightness of synaesthetic colours on brightness perception may also differ between projectors and associators. Therefore, we performed experiments on both projectors and associators to investigate the possibility.

## Method

### Participants

All experiments were carried out in accordance with the guidelines of the Declaration of Helsinki, and were approved by the Institutional Review Board of the University of Tokyo. Informed consent was obtained from all participants. Twenty-one Japanese grapheme-colour synaesthetes (17 females; mean age = 28.4, ranging from 19 to 49 years) and 40 Japanese non-synaesthetes (22 females; mean age = 22.4, ranging from 19 to 28 years) participated in this study. We were unable to equalize the distribution of age and gender between synaesthetes and non-synaesthetes: The group of synaesthetes were older (*t*(21) = 3.456, *p* = 0.002) and more female (χ^2^(1) = 4.022, *p* = 0.045) than non-synaesthetes. We consider this in the Results section. Although the first language of every synaesthete was Japanese, they reported experiencing synaesthetic colours when viewing English alphabet letters and Arabic numerals (digits), as well as when viewing Japanese characters. All non-synaesthetes reported having never experienced synaesthetic colours for any kind of grapheme. All participants had normal or corrected-to-normal visual acuity and colour vision. According to the Illustrated Synaesthetic Experience Questionnaire (ISEQ)^27^, synaesthetes were classified into ten associators and 11 projectors (none undetermined), based on the criterion defined by Skelton et al.^27^.

Synaesthetes participated in a colour matching task more than two weeks before participating in the main study. In a preliminary task, they were presented with each of the graphemes (26 English letters and ten Arabic numerals) in turn and were asked to select from an RGB 256^3^ colour palette a colour that most closely matched their synaesthetic experience. If the presented character elicited no synaesthetic colour, they were asked to select a ‘no colour’ option. At the beginning of the main study, they again engaged in the colour matching task, and we examined the consistency of their colour selections over time to confirm that they were genuine grapheme-colour synaesthetes^3^. The average colour distances in the CIE L*a*b* system for the colours selected for a given grapheme in the two tasks was calculated for each synaesthete.

According to Rothen, Seth, Witzel, and Ward^32^, when Euclidean distances in the CIE L*a*b* colour system were used to calculate colour distances, the cut-off value to discriminate synesthetes and non-synesthetes was 109.20. In their study, however, they had participants select colours three times for each grapheme within the same experiment, and they calculated the sum of three colour distances. Thus we divided the Rothen et al. cut-off score of 109.20 (the sum of three colour distances) by three and regarded the resulting 36.40 as our cut-off value. The consistency score of each synaesthete was below 36.40 (average = 25.18, *SD* = 4.78, min. = 13.96, max. = 31.61), indicating that the consistency of their colour selections was sufficient to regard them as synaesthetes. Because the colour selections were made at intervals of at least two weeks rather than within the same experiment^32^, the present method adequately demonstrates that every participant designated as a synaesthete in our study was indeed a genuine synaesthete.

### Apparatus

Stimuli were presented on a calibrated Sony colour display (CPD-G400J), using MATLAB (MathWorks) in conjunction with the Psychophysics Toolbox^33,34^. The pixel resolution of the colour CRT was 1280 × 1024 pixel and its refresh rate was 60 Hz. Participants viewed the monitor binocularly from a distance of 57 cm.

### Grapheme stimuli selection

For each synaesthete, we chose three graphemes (a ‘standard stimulus’, a ‘bright synaesthetic colour (SC) stimulus’, and a ‘dark SC stimulus’) based on the results of the colour matching task conducted at the beginning of the study. Although the first language of all participants was Japanese, we chose to use English alphabet letters and Arabic numerals as stimuli. We did this because many Japanese characters have complex forms and are spatially divided into multiple parts, and the orthographical properties of Japanese characters were thus more likely to interfere with brightness judgments than those of English alphabet letters and Arabic numerals.

All stimuli used in this experiment are illustrated in Supplementary Table S1. A standard stimulus and a bright SC stimulus evoked bright synaesthetic colours; these two stimuli were randomly selected from graphemes for which the luminance of the colours selected in the colour matching task was higher than 80 cd/m^2^. A dark SC stimulus evoked a dark synaesthetic colour; it was randomly selected from graphemes for which the luminance of the colour was lower than 20 cd/m^2^. To make the area occupied by graphemes constant, orthographically simple graphemes (‘I’, ‘J’, ‘L’, ‘T’, ‘1′, ‘7′) and complex graphemes (‘Q’, ‘W’) were not included as candidates. For two synaesthetes (an associator and a projector; S10 and S15 in Supplementary Table S1), no colours selected for each grapheme met the criteria noted above. Accordingly, for these individuals we selected a grapheme that evoked a synaesthetic colour with the highest luminance as a bright SC stimulus, a grapheme that evoked a colour with the second highest luminance as a standard stimulus, and a grapheme that evoked a colour with the lowest luminance as a dark SC stimulus.

Each non-synaesthete participant was randomly paired with one of the synaesthetes and was presented with the same set of stimuli as the paired synaesthete. These assignments were made such that the set of stimuli for each synaesthete was also presented to two non-synaesthetes.

### Procedure

**Figure 1:**
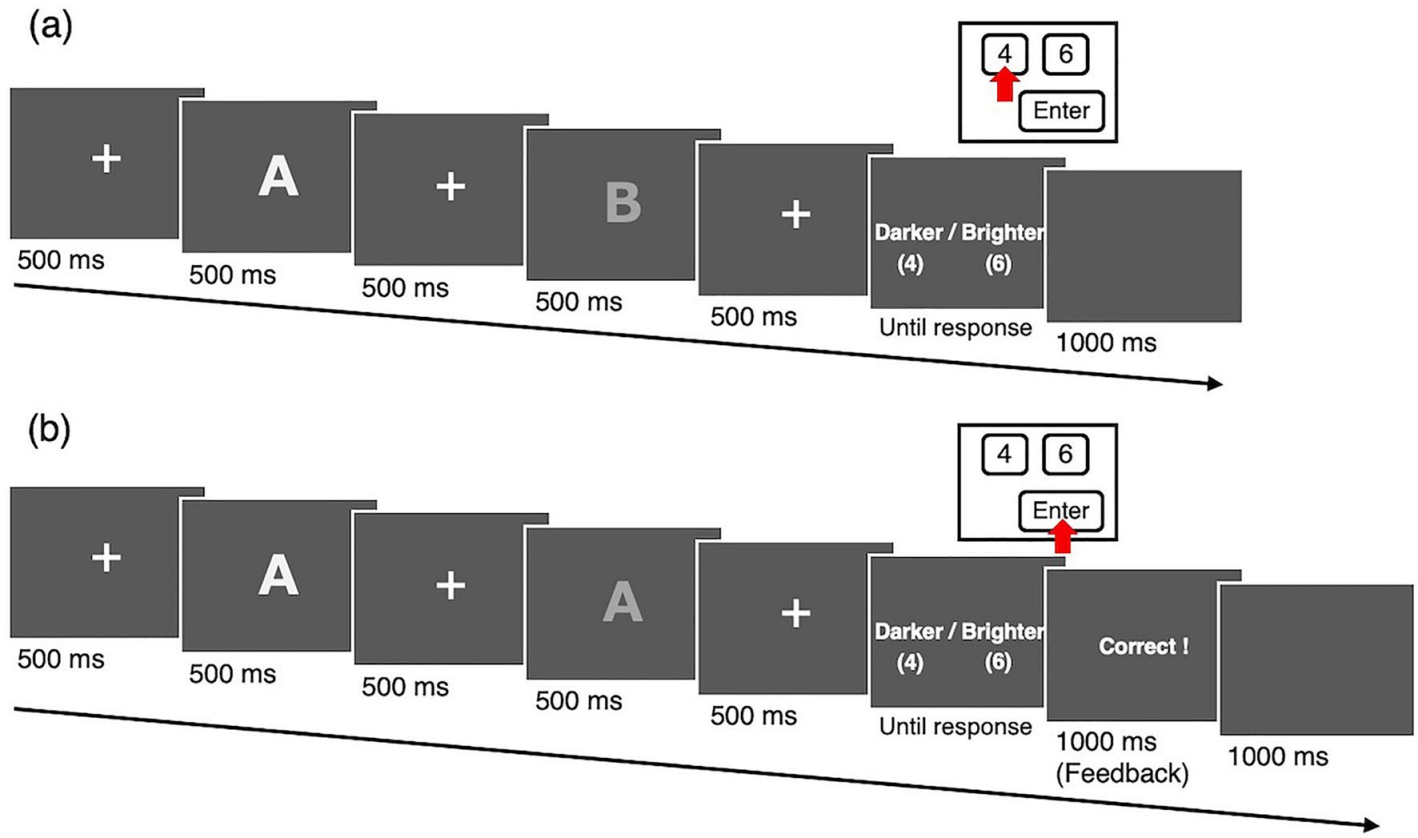
(**a**) Sequence of the brightness judgment task (main trials). Participants reported whether the second stimulus appeared brighter or darker than the first stimulus by pressing one of two computer keys (‘4′ or ‘6′ on the numeric keypad). (**b**) Sequence of dummy trials. Participants pressed the ‘Enter’ key on the numeric keypad irrespective of the perceived brightness of the two stimuli.

Participants performed a modified two-alternative forced choice task for brightness judgment (termed ‘main trials’; see Fig. 1a). At the beginning of each trial, a central fixation point was presented for 500 ms. Then each of the two grapheme stimuli was presented at the centre of the screen for 500 ms successively with an inter-stimulus interval of 500 ms. After the stimulus offset, participants reported whether the second stimulus appeared brighter or darker than the first stimulus by pressing one of two computer keys. If the participant was a synaesthete, we asked her/him to try to ignore her/his synaesthetic experience and report the apparent physical brightness.

One of the two stimuli was a standard stimulus, and the other was either a bright SC stimulus or a dark SC stimulus. All stimuli were grey (CIE [*x, y*] = [0.33, 0.33]) and were presented on a darker grey background (9.8 cd/m^2^; CIE [*x, y*] = [0.33, 0.33]). The luminance of the standard stimulus was fixed at 28.6 cd/m^2^, whereas the bright SC stimulus and the dark SC stimulus had ten levels of luminance (from 20.5 cd/m^2^ to 36.7 cd/m^2^; at 1.8 cd/m^2^ interval). Each of 20 types of comparison stimuli (two graphemes; the bright SC stimulus and the dark SC stimulus × ten levels of luminance) was used 40 times, in half of which the comparison stimulus was presented in advance of the standard stimulus. All stimuli were presented in a bold-faced Meiryo font and subtended 8.5° of visual angle.

During the main trials, participants were required to recognize graphemes, because synaesthetic colour experience is automatically triggered by grapheme identification and does not occur before the initial process of grapheme recognition^5,35^. So that the synaesthetes would experience synaesthetic colours in conjunction with brightness perception, they also performed ‘dummy trials’, which appeared with low probability (9.1%) among the main trials (Fig. 1b). In the dummy trials, the same grapheme stimuli, but with different luminances, were presented successively. Although the response screens for the dummy trials were the same as those for the main trials, participants were required to press a key different from that used for the brightness judgments. In order to produce a correct response in the dummy trials, participants had not only to compare the brightness of grapheme stimuli but also to identify them. Feedback showing whether the key press was correct or incorrect was displayed after each response. There were 80 dummy trials in total, and the standard stimuli were presented in 40 trials, the bright SC stimuli were presented in 20 trials, and the dark SC stimuli were presented in 20 trials continuously.

The task comprised 880 trials in total, and the order of trials was completely randomized for each participant. Forty-four practice trials, consisting of four dummy trials and 40 main trials, preceded the experimental trials.

## Results

### Dummy trial

The mean error rate on the dummy trials for all participants was 4.80% (*SD* = 5.74). The mean error rate on the dummy trials was 4.37% (*SD* = 6.91) for synaesthetes and 5.03% (*SD* = 5.00) non-synaesthetes, a non-significant difference (*t*(31) = 0.38, *p* = 0.706). However, the error rate on the dummy trials for one synaesthete (a projector, S11 in Supplementary Table S1 was 31.75%, which substantially exceeds 3 *SD* from the mean of each participant. We regarded this participant as having failed to recognize graphemes during the brightness judgment task, and we excluded his data from all subsequent analyses. Because we decided to exclude his data before non-synaesthetes participated in this study, no non-synaesthete was subsequently paired with him to perform the brightness judgment task using the three grapheme stimuli.

### Data analysis

**Figure 2:**
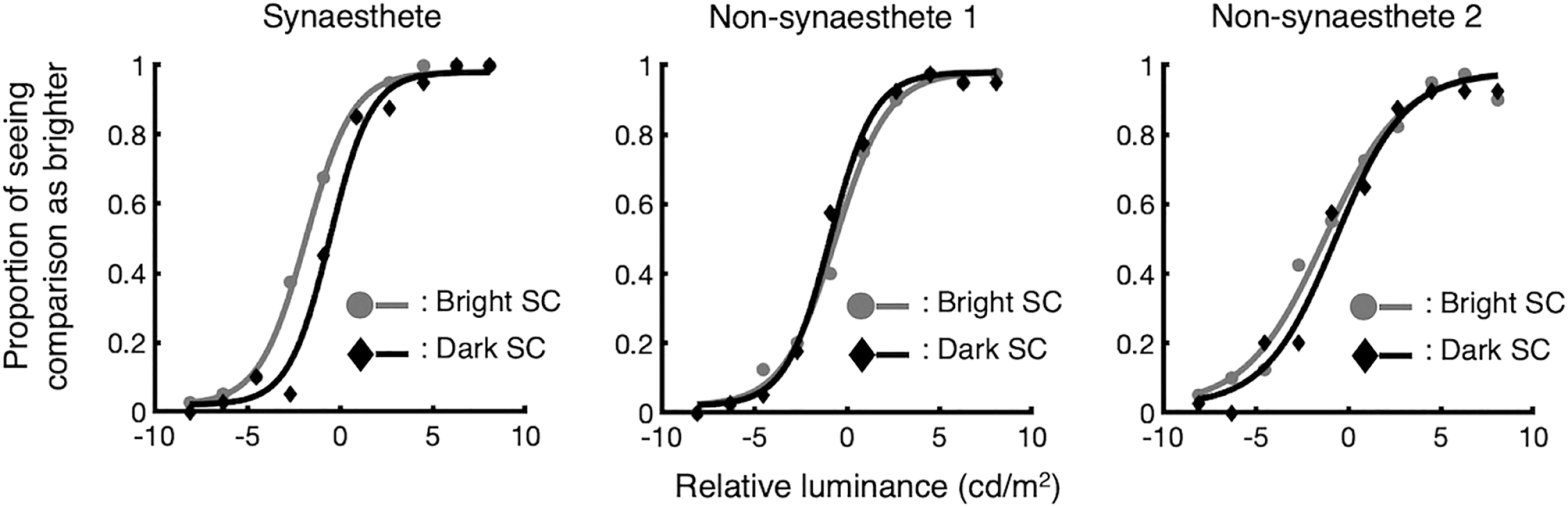
Examples of psychometric functions. Left: one projector-type synaesthete (S18 in Supplementary Table S1). Centre and right: two non-synaesthetes who were presented with the identical set of stimuli as the synaesthete. Shifts of the curve toward the left indicate that the comparison stimulus was judged as brighter than the standard stimulus, even when the actual luminance was darker.

We obtained two psychometric functions by fitting the proportions of the comparison stimuli (the bright SC stimulus and the dark SC stimulus) that were judged as brighter than the standard stimuli for each participant. The data were fitted with a logistic distribution with a lapse rate of 0.02 by maximum likelihood estimation, using the Palamedes Toolbox extensions for MATLAB^36^. Exemplar psychometric functions (one projector-type synaesthete, S18 in Supplementary Table S1, and two non-synaesthetes who were presented with an identical set of stimuli as the synaesthete) are shown in Fig. 2. The midpoint of the function was regarded as the point of subjective equality (PSE) and the slope around the PSE as the index of sensitivity.

For one synaesthete (an associator, S7 in Supplementary Table S1), the proportion of dark SC stimuli that was judged as brighter never reached 50%, and the PSE could not be defined within the scope of the luminance of the comparison stimuli. Thus, we excluded the data of this synaesthete, and the data of two non-synaesthetes presented with the identical set of stimuli as this synaesthete from all subsequent analyses.

### Brightness judgment task

**Figure 3:**
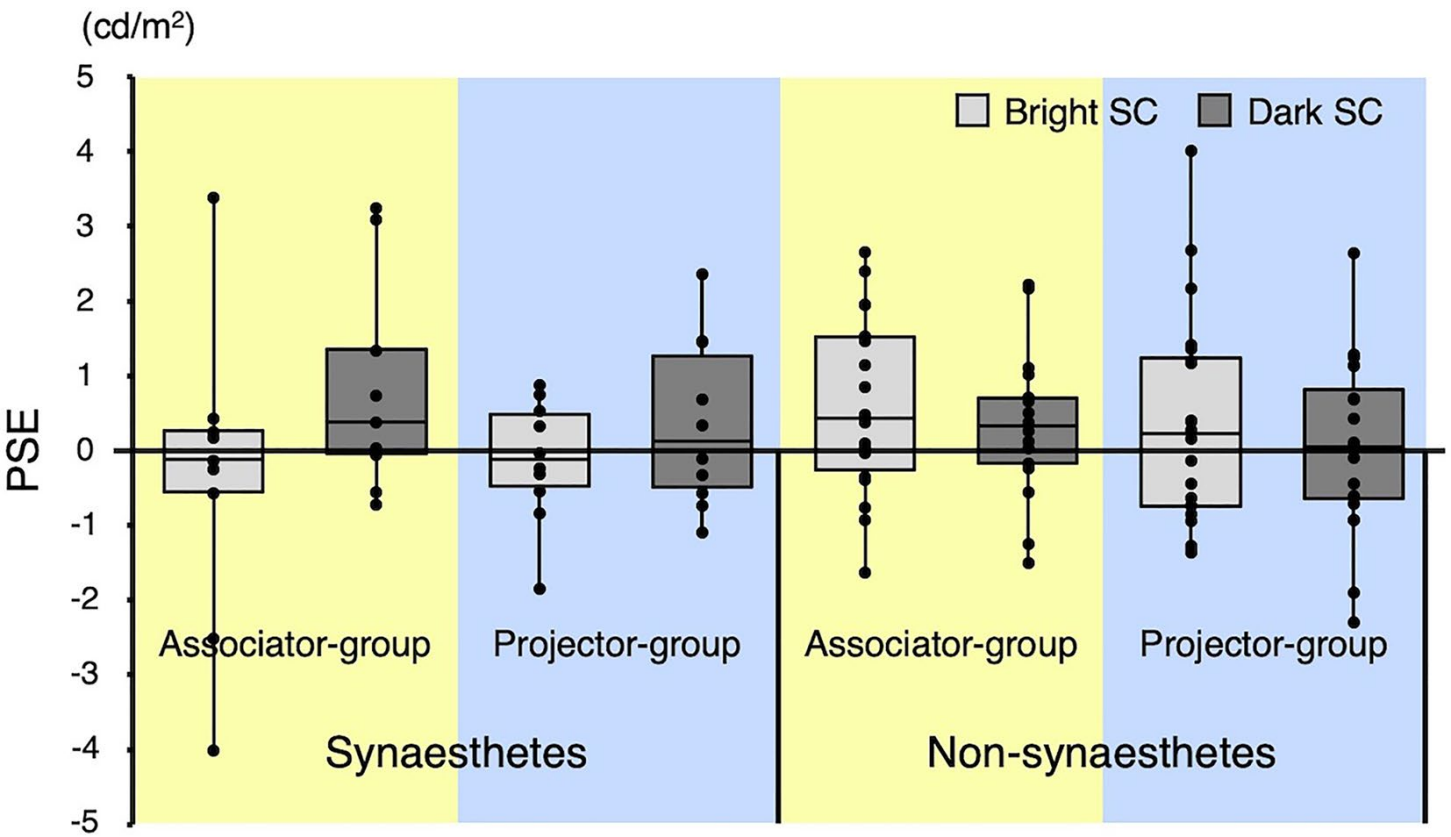
Boxplots of PSE data as a function of subject type (synaesthete or non-synaesthete), stimulus set group (associator group or projector group), and comparison stimulus type (bright SC stimulus or dark SC stimulus). Boxes represent interquartile ranges and central horizontal lines are medians. For each box, the vertical line indicates maximum and minimum values. Individual data are plotted together with the boxplots.

To make possible a comparison of associators and projectors, we divided participants into two groups based on the sets of stimuli they were presented with: the associator-type synaesthetic participants and the non-synaesthetic participants that were presented with the identical set of stimuli as associators were regarded as the ‘associator group’, and the projector-type synaesthetic participants and the non-synaesthetic participants that were presented with the identical set of stimuli as projectors in the ‘projector group’. We conducted a three-way mixed-design ANOVA on PSE (Fig. 3), with factors of subject type (synaesthete or non-synaesthete) and stimulus set group (associator group or projector group) as between-participants factors and comparison stimulus type (bright SC stimulus or dark SC stimulus) as a within-participants factor. No main effects were significant (subject type: *F*(1, 53) = 0.29, *p* = 0.590, *η*^2^ = 0.004; stimulus set group: *F*(1, 53) = 0.39, *p* = 0.536, *η*^2^ = 0.005; comparison stimulus type: *F*(1, 53) = 0.03, *p* = 0.848, *η*^2^ = 0.001). However, the interaction between subject type and comparison stimulus type was significant (*F*(1, 53) = 8.15, *p* = 0.006, *η*^2^ = 0.044), indicating that the effect of comparison stimulus type differed between synaesthetes and non-synaesthetes. The PSE value for bright SC and dark SC stimuli in synaesthetes was − 0.22 (*SE* = 0.34) and 0.59 (*SE* = 0.29), respectively, whereas the PSE value for bright SC and dark SC stimuli in non-synaesthetes was 0.50 (*SE* = 0.22) and 0.19 (*SE* = 0.17), respectively. We conducted follow-up, simple effects tests on the interaction, which indicated that the effect of comparison stimulus type was neither significant for synaesthetes (*F*(1, 17) = 3.70, *p* = 0.071, *η*^2^ = 0.087) nor for non-synaesthetes (*F*(1, 36) = 2.78, *p* = 0.104, *η*^2^ = 0.017). Moreover, the simple effect of subject type was neither significant for bright SC stimulus (*F*(1, 53) = 3.46, *p* = 0.068, *η*^2^ = 0.061) nor for dark SC stimulus (*F*(1, 53) = 1.60, *p* = 0.212, *η*^2^ = 0.029). No other interactions were significant (subject type × stimulus set group: *F*(1, 53) = 0.04, *p* = 0.848, *η*^2^ = 0.001; stimulus set group × comparison stimulus type: *F*(1, 53) = 0.88, *p* = 0.351, *η*^2^ = 0.005; subject type × stimulus set group × comparison stimulus type: *F*(1, 53) = 0.68, *p* = 0.414, *η*^2^ = 0.004). The absence of significant three-factor interactions suggests that there was no significant difference between associators and projectors in the size of the effect of stimulus type. Note that, as mentioned in “Method”, synaesthetes were older and more female than non-synaesthetes. However, these differences were not expected to affect the PSE results, because PSEs did not vary by age or gender for either the synaesthetes or non-synaesthetes (see Supplementary Analysis for details).

**Figure 4:**
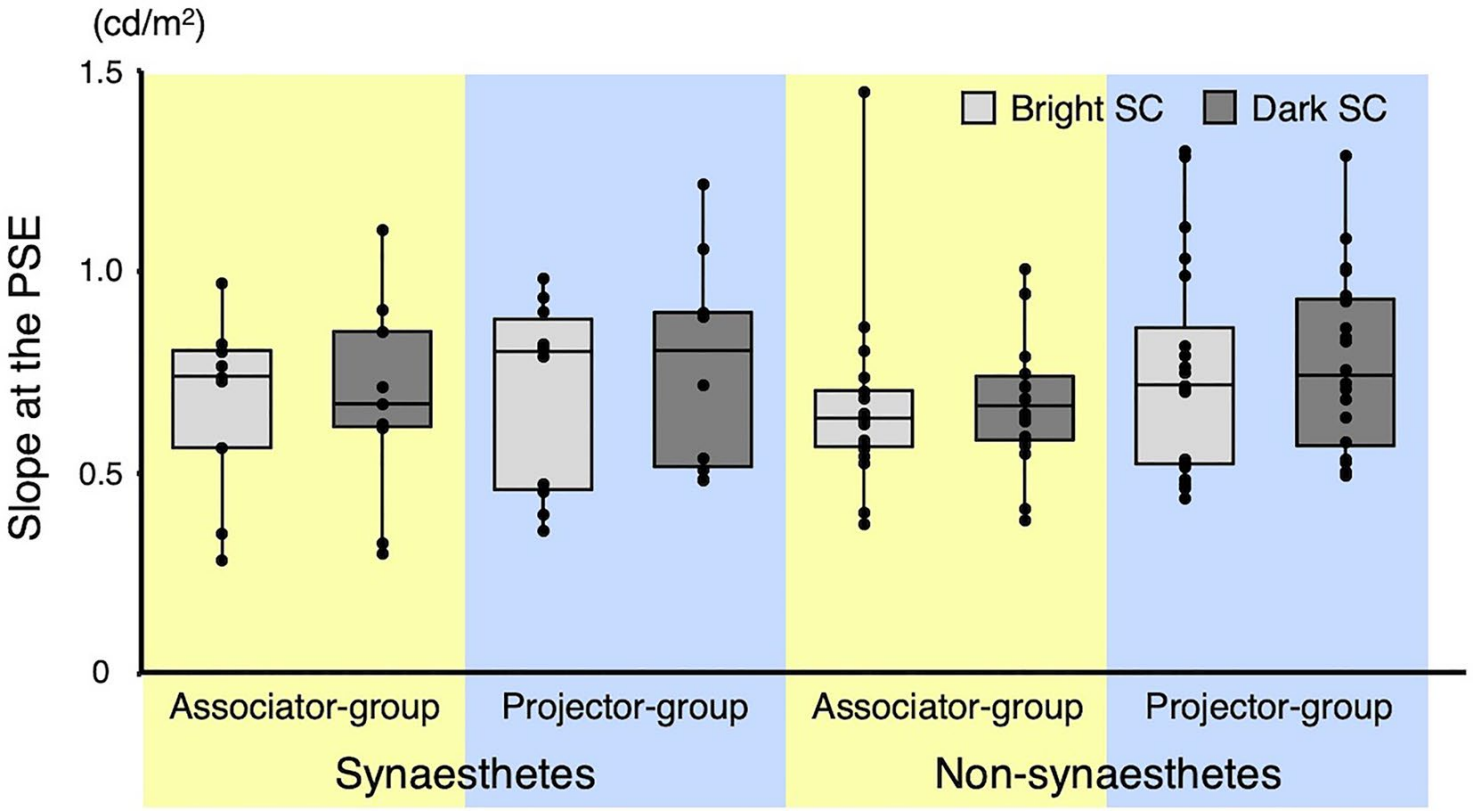
Boxplots of slope data at the PSE as a function of subject type (synaesthete or non-synaesthete), stimulus set group (associator group or projector group), and comparison stimulus type (bright SC stimulus or dark SC stimulus). Boxes represent interquartile ranges and central horizontal lines are medians. For each box, the vertical line indicates maximum and minimum values. Individual data are plotted together with the boxplots.

We also conducted a three-way mixed-design ANOVA on slopes by subject type, stimulus group set, and comparison stimulus type (Fig. 4). No significant main or interaction effects were found (main effects: subject type: *F*(1, 53) = 0.08, *p* = 0.784, *η*^2^ = 0.001; stimulus set group: *F*(1, 53) = 1.45, *p* = 0.234, *η*^2^ = 0.022; comparison stimulus type: *F*(1, 53) = 1.19, *p* = 0.281, *η*^2^ = 0.003; interactions: subject type × stimulus set group: *F*(1, 53) = 0.08, *p* = 0.785, *η*^2^ = 0.001; subject type × comparison stimulus type: *F*(1, 53) = 0.33, *p* = 0.570, *η*^2^ = 0.001; stimulus set group × comparison stimulus type: *F*(1, 53) = 0.48, *p* = 0.491, *η*^2^ = 0.001; subject type × stimulus set group × comparison stimulus type: *F*(1, 53) = 0.37, *p* = 0.543, *η*^2^ = 0.001), indicating that synaesthetic colour experience did not affect the discriminability of luminance.

### Brightness change correlates with luminance of the reported synaesthetic colours

The above results show that the effect of the comparison stimulus type differs between synaesthetes and non-synaesthetes, indicating the possibility that the luminance of synaesthetic colours experienced by synaesthetes modulated the perceived brightness of graphemes. We also examined whether the number of changes in the perceived brightness of graphemes for each synesthete correlated with the difference in luminance between the synaesthetic colour of the standard stimulus and that of the comparison stimuli (Bright SC stimulus and Dark SC stimulus). To control the influence of the form of the grapheme stimuli on brightness perception, for each synaesthete we defined the modified PSE as the PSE of that synaesthete minus the mean of the PSEs of the two non-synaesthetes, who had been presented with the same set of stimuli as the paired synaesthete in the brightness judgment task.

**Figure 5:**
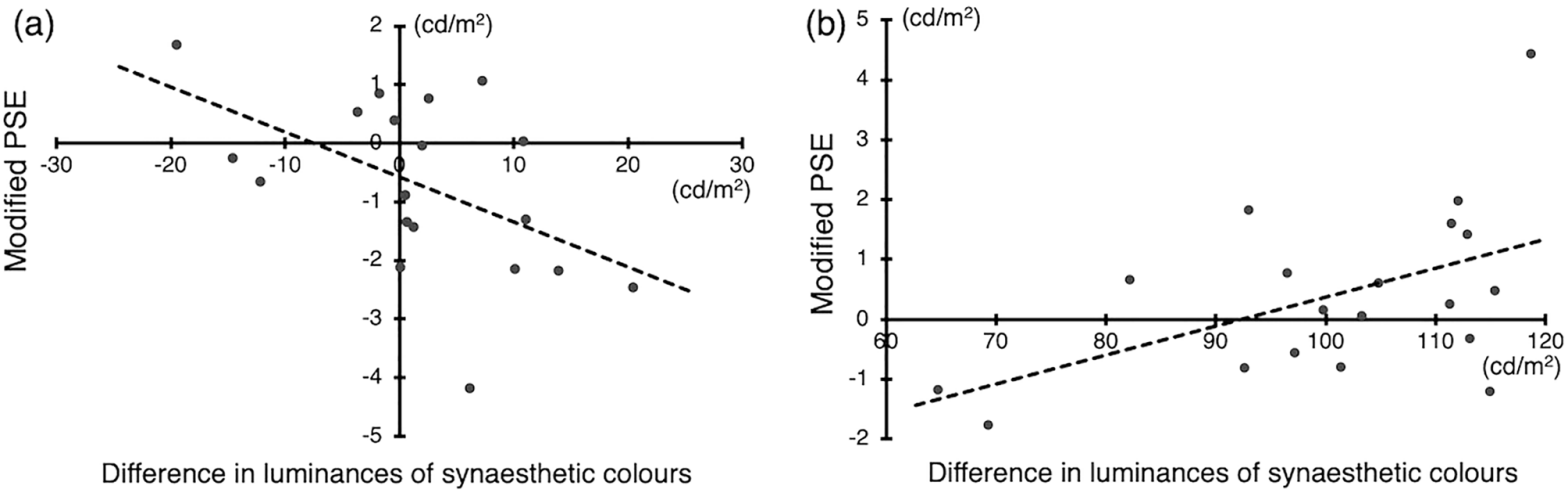
Relationship between the modified PSE of a comparison stimulus in synaesthetes and the difference in luminance between the synaesthetic colour of the comparison stimulus and that of the standard stimulus. A larger positive PSE value indicates that the comparison stimulus is physically perceived as darker than the standard stimulus. (**a**) Comparison of standard and bright SC stimuli. A larger positive difference in luminance of synaesthetic colours indicates that the synaesthetic colour of the bright SC stimulus is perceived as brighter than that of the standard stimulus. (**b**) Comparison of standard and dark SC stimuli. A larger positive difference in luminance of synaesthetic colours indicates that the synaesthetic colour of the dark SC stimulus is perceived as darker than that of the standard stimulus.

There was a significant negative correlation between the modified PSE of the bright SC stimulus and the difference in luminance between the synaesthetic colour of the bright SC stimulus and that of the standard stimulus (*r* = − 0.51, *t*(17) = 2.439, *p* = 0.026; see Fig. 5a). We also found a significant positive correlation between the modified PSE of the dark SC stimulus and the difference in luminance between the synaesthetic colour of the standard stimulus and that of the dark SC stimulus (*r* = 0.51, *t*(17) = 2.448, *p* = 0.026; see Fig. 5b). These results demonstrate that the brighter/darker a synaesthete perceives the synaesthetic colour of a comparison stimulus to be as compared to a standard stimulus, the brighter/darker the physical brightness of the comparison stimulus is perceived to be, supporting our hypothesis that experienced synaesthetic colour modulates the perceived brightness of graphemes.

## Discussion

The result of the brightness judgment task demonstrated a significant interaction between subject type (synaesthete or non-synaesthete) and comparison stimulus type (bright SC stimulus or dark SC stimulus), indicating that the effect of comparison stimulus type differs between synaesthetes and non-synaesthetes. However, the stimuli did not elicit synaesthetic colours in non-synesthetes. Therefore, this difference might be caused by differences in the brightness of synaesthetic colours between the two comparison stimuli for synesthetes. Furthermore, the size of the PSE shift in luminance between standard stimulus and comparison stimulus for each synaesthete was correlated with the difference in luminance between the synaesthetic colour of the standard stimulus and that of the comparison stimulus. These results suggest that the graphemes’ apparent physical brightness was modulated by the synaesthetic colours they elicited.

As noted above, we were not able to find a significant simple effect of stimulus type in synesthetes. One possible reason is that the orthographical properties of the grapheme stimuli may have been confounded in the brightness judgment task. In non-synesthetes, the PSE values of the bright SC stimuli tended to be higher than those of the dark SC stimuli, although not significantly different (see Fig. 3), suggesting that the bright SC stimuli tended to be perceived as darker than the dark SC stimuli. Because non-synesthetes do not perceive any synaesthetic colours for grapheme stimuli, this tendency may be due to differences in orthographical properties (e.g., contrast with the background) of grapheme stimuli used in the task. Thus, in synaesthetes, the orthographical properties of the grapheme stimuli may have had an effect on brightness judgments opposite to their synaesthetic colours.

Another possible reason is that standard stimuli, as well as bright SC stimuli, elicited bright synaesthetic colours for the synaesthetes. Although the synaesthetic colour of a dark SC stimulus was darker than that of a standard stimulus for every synaesthete in the brightness judgment task, the luminance of the synaesthetic colour of a bright SC stimulus was not necessarily higher than that of a standard stimulus. If the grapheme stimuli used in the brightness judgment task had been presented such that the luminance of the synaesthetic colour of a standard stimulus was halfway between that of a bright SC stimulus and that of a dark SC stimulus, the difference in perceived brightness between the two comparison stimuli may have been demonstrated more clearly by synaesthetes.

Synaesthetic colour experience did not affect the slopes around the PSEs (i.e., the discriminability of luminance). The finding that the discrimination threshold did not differ between synaesthetes and non-synaesthetes suggests that synaesthetes could ignore their synaesthetic experience and report apparent physical brightness during the brightness judgment task, just as non-synaesthetes did. This demonstrates that synaesthetes have the cognitive ability to separate perception of real brightness (and colours) from the experience of synaesthetic colours. Note, however, that for only one synaesthete (an associator, S7 in Supplementary Table S1), the proportion of dark SC stimuli that were judged as brighter never reached 50%, indicating that she could not separate real brightness perception from her synaesthetic colour sensation (see “Data analysis”).

Our finding that synaesthetic colour experience alters brightness perception supports the possibility that modulatory feedback from the V4 colour area to the low-level perceptual process^20,21^ co-occurs with synaesthetic colour experience. Although we cannot conclude definitively that the alteration of real brightness perception is triggered by neural activation in V4, which accompanies synaesthetic colour experience^14–17^, we have two reasons to suspect that V4 activation may cause the modulation of brightness perception.

The first reason is that modulation in real brightness perception was observed in both projectors and associators. As mentioned above, previous research in neuroscience and psychology has suggested that there are differences between projectors and associators in the processes that cause synaesthetic colour experience^27–30^. The results of our study suggest that the process that causes synaesthetic colours may not affect brightness perception, but that activation of V4 as a result of the processing may cause synaesthetic colour experience and at the same time alter brightness perception.

The second reason is that a comparison between earlier research and the present study suggests that the experience of synaesthetic colour with V4 activation plays an important role in the modulation of normal visual processing. Only one prior psychophysical study^11^ has investigated whether synaesthetic colour experience modulates colour perception. Hong and Blake^11^ conducted three experiments aimed at clarifying the interaction between synaesthetic colours and colour and brightness perception. One experiment tested three synaesthetes on a task designed to examine the influence of synaesthetic colours on perceived colours of graphemes. In this task, synaesthetes used a cancellation technique^37^ to adjust the colours of two chromatic graphemes that induced synaesthetic colours of red and green, as well as a neutral symbol that induced no synaesthetic colour, until all three items appeared neither reddish nor greenish, an appearance referred to as equilibrium yellow. The results showed that the red/green ratios producing equilibrium yellow did not differ among the three conditions, indicating that synaesthetic colour experience does not modulate colour (at least hue) perception. In the task conducted by Hong and Blake^11^, however, synaesthetes only judged the real colours of graphemes and were not required to identify the graphemes or to pay attention to their synaesthetic colours. Synaesthetic colour experience is automatically triggered by grapheme identification and does not occur before the initial process of grapheme recognition^5,35^. Therefore, it is unclear whether synaesthetes sustained their synaesthetic colour experience throughout the hue cancellation task. On the other hand, in the present study, we had synaesthetes perform dummy trials and had them recognize graphemes. If the synaesthetic colour experience triggered by grapheme recognition modulated brightness perception, the difference between the results of the previous study^11^ and those of our study may be explained by the absence or presence of V4 activation.

Our findings that synaesthetic colour modulates brightness perception are consistent with previous findings that visual memory affects colour appearance^38–40^. These studies have shown that the typical colours of objects can modulate colour appearance; for example, at the point where a banana was physically achromatic, it still appeared slightly yellowish^38^. These studies have suggested that people apply prior knowledge about the natural colour of objects in order to perceive their constant surface colours despite large changes in illumination. Our results suggest that the synaesthetic colour of a grapheme functions as prior knowledge of the grapheme as effectively as does the typical colour of an object^38–40^, and when a synaesthetic colour experience is triggered, this prior knowledge modulates the perceived brightness of the grapheme to the brightness of the synaesthetic colour. For synaesthetes, the perceptual and cognitive consequences of this modulation can contribute to colour-guided grapheme learning. A recent line of research on grapheme-colour synaesthesia and grapheme learning suggests that synaesthetic colours function as ‘discriminating markers’ of graphemes, facilitating grapheme discrimination and learning in childhood^22,41–43^. Based on these findings, it is possible that the modulatory feedback transmitted from V4, the neural activation of which accompanies synaesthetic colour experience, to the early stages of visual processing makes brightness perception constant, and thus enhances the marking potential of synaesthetic colours for grapheme discrimination.

## Supplementary information

Supplementary Information 1.

## Data Availability

The data that support the current study are available at https://osf.io/9zwqv/.

## References

[1] Simner J (2007). Beyond perception: Synaesthesia as a psycholinguistic phenomenon. Trends Cogn. Sci..

[2] Rich AN, Bradshaw JL, Mattingley JB (2005). A systematic, large-scale study of synaesthesia: Implications for the role of early experience in lexical-colour associations. Cognition.

[3] Eagleman DM, Kagan AD, Nelson SS, Sagaram D, Sarma AK (2007). A standardized test battery for the study of synesthesia. J. Neurosci. Methods.

[4] Dixon M, Smilek D, Cudahy C, Merikle PM (2000). Five plus two equals yellow. Nature.

[5] Mattingley JB, Rich AN, Yelland G, Bradshaw JL (2001). Unconscious priming eliminates automatic binding of colour and alphanumeric form in synaesthesia. Nature.

[6] Dixon MJ, Smilek D, Merikle PM (2004). Not all synaesthetes are created equal: Projector versus associator synaesthetes. Cogn. Affect. Behav. Neurosci..

[7] Ward J, Li R, Salih S, Sagiv N (2007). Varieties of grapheme-colour synaesthesia: A new theory of phenomenological and behavioural differences. Conscious Cogn..

[8] Hubbard EM, Manohar S, Ramachandran VS (2006). Contrast affects the strength of synesthetic colors. Cortex.

[9] Witthoft N, Winawer J (2006). Synesthetic colors determined by having colored refrigerator magnets in childhood. Cortex.

[10] Erskine H, Mattingley JB, Arnold DH (2013). Synaesthesia and colour constancy. Cortex.

[11] Hong SW, Blake R (2008). Early visual mechanisms do not contribute to synesthetic color experience. Vis. Res..

[12] Nijboer TCW, Gebuis T, te Pas SF, van der Smagt MJ (2011). Interactions between colour and synaesthetic colour: an effect of simultaneous colour contrast on synaesthetic colours. Vis. Res..

[13] McErlean ABJ, Banissy MJ (2017). Color processing in synesthesia: What synesthesia can and cannot tell us about mechanisms of color processing. Top. Cogn. Sci..

[14] Hubbard EM, Arman AC, Ramachandran VS, Boynton GM (2005). Individual differences among grapheme-color synesthetes: brain-behavior correlations. Neuron.

[15] Rouw R, Scholte HS (2007). Increased structural connectivity in grapheme-color synesthesia. Nat. Neurosci..

[16] Sperling JM, Prvulovic D, Linden DE, Singer W, Stirn A (2006). Neuronal correlates of colour-grapheme synaesthesia: a fMRI study. Cortex.

[17] van Leeuwen, T. M., Petersson, K. M., & Hagoort, P. Synaesthetic colour in the brain: Beyond colour areas. A functional magnetic resonance imaging study of synaesthetes and matched controls. *PLoS ONE* **5**, e12074. 10.1371/journal.pone.0012074 (2010). 10.1371/journal.pone.0012074 PMC2919410 20711467

[18] Bartels A, Zeki S (2000). The architecture of the colour centre in the human visual brain: New results and a review. Eur. J. Neurosci..

[19] Hupé JM, Bordier C, Dojat M (2012). The neural bases of grapheme-color synesthesia are not localized in real color-sensitive areas. Cereb. Cortex.

[20] Gilbert CD, Li W (2013). Top-down influences on visual processing. Nat. Rev. Neurosci..

[21] Rockland KS, Saleem KS, Tanaka K (1994). Divergent feedback connections from areas V4 and TEO in the macaque. Visual Neurosci..

[22] Asano M, Yokosawa K (2013). Grapheme learning and grapheme-color synesthesia: Toward a comprehensive model of grapheme-color association. Front. Hum. Neurosci..

[23] Hung W-Y, Simner J, Shillcock R, Eagleman DM (2014). Synaesthesia in Chinese characters: the role of radical function and position. Conscious Cogn..

[24] Watson MR, Akins KA, Enns JT (2012). Second-order mappings in grapheme-color synesthesia. Psychon. B. Rev..

[25] Beeli G, Esslen M, Jäncke L (2007). Frequency correlates in grapheme-colour synaesthesia. Psychol. Sci..

[26] Smilek D, Carriere JSA, Dixon MJ, Merikle PM (2007). Grapheme frequency and color luminance in grapheme-color synaesthesia. Psychol. Sci..

[27] Skelton R, Ludwig C, Mohr C (2009). A novel, illustrated questionnaire to distinguish projector and associator synaesthetes. Cortex.

[28] Rouw R, Scholte HS (2010). Neural basis of individual differences in synesthetic experiences. J. Neurosci..

[29] van Leeuwen TM, den Ouden HEM, Hagoort P (2011). Effective connectivity determines the nature of subjective experience in grapheme-color synesthesia. J. Neurosci..

[30] Brang D, Rouw R, Ramachandran VS, Coulson S (2011). Similarly shaped letters evoke similar colors in grapheme-color synesthesia. Neuropsychologia.

[31] Hamada D, Yamamoto H, Saiki J (2017). Multilevel analysis of individual differences in regularities of grapheme-color associations in synesthesia. Conscious Cogn..

[32] Rothen N, Seth AK, Witzel C, Ward J (2013). Diagnosing synaesthesia with online colour pickers: maximising sensitivity and specificity. J. Neurosci. Methods.

[33] Brainard, D. H. The psychophysics toolbox. *Spatial Vis.* **10**, 433–436 (1997). 9176952

[34] Pelli DG (1997). The videotoolbox software for visual psychophysics: transforming numbers into movies. Spatial Vis..

[35] Brang D, Hubbard EM, Coulson S, Huang M, Ramachandran VS (2010). Magnetoencephalography reveals early activation of V4 in grapheme-colour synaesthesia. NeuroImage.

[36] Prins N, Kingdom FAA (2018). Applying the model-comparison approach to test specific research hypotheses in psychophysical research using the Palamedes Toolbox. Front. Psychol..

[37] Hurvich LM, Jameson D (1957). An opponent-process theory of color vision. Psychol. Rev..

[38] Hansen T, Olkkonen M, Walter S, Gegenfurtner KR (2006). Memory modulates color appearance. Nat. Neurosci..

[39] Olkkonen, M., Hansen, T., & Gegenfurtner, K. R. Color appearance of familiar objects: Effects of object shape, texture, and illumination changes. *J. Vis.* **8**(5), 1–16. 10.1167/8.5.13. (2008). 10.1167/8.5.13 18842084

[40] Witzel, C., Olkkonen, M., & Gegenfurtner, K. R. A bayesian model of the memory colour effect. *i-Perception* **9,** 1–16. 10.1177/2041669518771715 (2018). 10.1177/2041669518771715 PMC5946617 29760874

[41] Uno K, Asano M, Kadowaki H, Yokosawa K (2020). Grapheme-color associations can transfer to novel graphemes when synesthetic colors function as grapheme “discriminating markers”. Psychon. Bull. Rev..

[42] Watson MR, Akins KA, Spiker C, Crawford L, Enns JT (2014). Synesthesia and learning: a critical review and novel theory. Front. Hum. Neurosci..

[43] Watson MR, Chromý J, Crawford L, Eagleman DM, Enns JT, Akins KA (2017). The prevalence of synaesthesia depends on early language learning. Conscious Cogn..

