# Supplementary material for: Apparent physical brightness of graphemes is altered by their synaesthetic colour in grapheme-colour synaesthetes
Source: Sci Rep. 2020 Nov 18;10:20134. doi: 10.1038/s41598-020-77298-2 (PMC7674506; doi:10.1038/s41598-020-77298-2)
Supplement: Supplementary file 1 — Supplementary Information 1. [file 41598_2020_77298_MOESM1_ESM.docx]

**Supplementary information**

**Apparent physical brightness of graphemes is altered by their synaesthetic colour in grapheme-colour synaesthetes**

**Kyuto Uno^1,*^ and Kazuhiko Yokosawa^1^**

^1^Department of Psychology, The University of Tokyo, Tokyo, 113-0033, Japan.

*uno@l.u-tokyo.ac.jp

Supplementary Table S1. Stimuli presented in the experiment and their synaesthetic colours for each synaesthete.

*Note*. 'x' and 'y' represent values of each synaesthetic colour in the CIE 1931 chromaticity diagram, and 'L' denotes luminance. Grapheme stimuli were displayed in the corresponding synaesthetic colour.

**Supplementary Analysis**

In our study, the group of synaesthetic participants were older (*t*(21) = 3.456, *p* = .002) and more female (χ^2^(1) = 4.022, *p* = .045) than the group of non-synaesthetes. We performed post hoc analyses to determine if age and gender affected judgments about the relative brightness of grapheme stimuli in the brightness judgment task. These analyses were performed for both synaesthetes and non-synaesthetes.

There were no significant correlations between age and PSE in synaesthetes (age × PSE of bright SC stimulus: *r* = -.31, *t*(17) = 1.366, *p* = .190; age × PSE of dark SC stimulus: *r* = -.26, *t*(17) = 1.127, *p* = .275; age × difference between these PSEs: *r* = .069, *t*(17) = 0.285, *p* = .779) or in non-synaesthetes (age × PSE of bright SC stimulus: *r* = .14, *t*(36) = 0.867, *p* = .392; age × PSE of dark SC stimulus: *r* = .27, *t*(36) = 1.687, *p* = .100; age × difference between these PSEs: *r* = .089, *t*(36) = 0.534, *p* = .597), indicating that age would not have an effect on the brightness judgments. Furthermore, there were no significant differences in PSE between females and males among synaesthetes (PSE of bright SC stimulus: *t*(17) = 1.489, *p* = .155; PSE of dark SC stimulus: *t*(17) = 1.926, *p* = .071; difference between these PSEs: *t*(17) = 1.266, *p* = .333) or non-synaesthetes (PSE of bright SC stimulus: *t*(36) = 1.415, *p* = .168; PSE of dark SC stimulus: *t*(36) = 0.185, *p* = .854; difference between these PSEs: *t*(36) = 1.503, *p* = .145), indicating that gender would not have an effect on brightness judgments. Based on these results, it is likely that the differences in age and gender did not affect the results for PSE.
